# Panicle Angle is an Important Factor in Tef Lodging Tolerance

**DOI:** 10.3389/fpls.2020.00061

**Published:** 2020-02-13

**Authors:** Regula Blösch, Sonia Plaza-Wüthrich, Pierre Barbier de Reuille, Annett Weichert, Anne-Lise Routier-Kierzkowska, Gina Cannarozzi, Sarah Robinson, Zerihun Tadele

**Affiliations:** ^1^Institute of Plant Sciences, University of Bern, Bern, Switzerland; ^2^Institut de Recherche en Biologie Végétale, University of Montreal, Montréal, QC, Canada; ^3^The Sainsbury Laboratory, University of Cambridge, Cambridge, United Kingdom

**Keywords:** *Eragrostis tef*, lodging, mechanical property, modeling, stem bending, tef

## Abstract

Tef [*Eragrostis tef* (Zucc.) Trotter] is an important crop in the Horn of Africa, particularly in Ethiopia, where it is a staple food for over 60 million people. However, the productivity of tef remains extremely low in part due to its susceptibility to lodging. Lodging is the displacement of the plant from the upright position, and it is exacerbated by rain, wind and the application of fertilizer. In order to address the issue of global food security, especially in the Horn of Africa, greater insight into the causes of tef lodging is needed. In this study, we combine modeling and biomechanical measurements to compare the properties relating to lodging tolerance in high yielding, improved tef genotypes, and lower yielding natural landraces. Our results indicate that the angle of the panicle contributes to the likelihood of lodging in tef. Varieties with compact panicles and reduced height had increased lodging resistance compared to the other varieties. By comparing different varieties, we found that overall, the landraces of tef lodged less than improved varieties. We constructed a model of stem bending and found that panicle angle was an important determinant of the amount of lodging. The findings from this study provide key information to those involved in tef improvement, especially those interested in lodging tolerance.

## Introduction

As the most important cereal crop in Ethiopia, tef [*Eragrostis tef* (Zucc.) Trotter] is annually cultivated on about 30% of the total land devoted to cereal crops (CSA, 2015). Tef is critical to food security in the Horn of Africa, where over 60 million people in Ethiopia alone consume the grain as a staple food. Tef is a resilient crop that performs better than other cereals under local conditions including drought, waterlogging and poor soil; however, its productivity is extremely low. The low productivity of tef is in part due to lodging (Assefa et al., 2011b), which is the permanent displacement of the stem from the upright position (Fischer and Stapper, 1987; Berry et al., 2004). In cereal crops, three types of lodging have been identified: (i) *culm bending-type lodging* which occurs when plants fail to resist bending pressure, as is often seen in the upper internodes of rice affected by strong winds and rain; (ii) *culm breaking-type lodging* that usually affects the lower internodes when there is excessive bending pressure at the upper internodes; and (iii) *root lodging* which happens when the roots give in to the weight of the above-ground parts (Hirano et al., 2017). Lodging angle captures the effects of culm breaking, culm bending and root lodging.

Lodging substantially affects tef productivity since the plant has a tall and slender stem. In addition, when fertilizer is applied to increase the productivity of the crop, stems of tef grow taller and become even more susceptible to lodging, resulting in significantly reduced quantity and quality of grain and straw. The average yield reduction due to lodging was estimated to be 17% (Ketema, 1993). Although different types of lodging were reported for tef, root lodging was dominant over the stem lodging (Van Delden et al., 2010). The introduction of semidwarf varieties of rice and wheat during the green revolution greatly reduced culm bending-type lodging and increased productivity (Hedden, 2003; Hirano et al., 2017). However, due to the high value of the tef straw as a livestock feed, breeding for a significant reduction in plant height might have little acceptance by the farmers (Yami, 2013).

Recent studies showed that semidwarf tef plants were achieved using inhibitors of gibberellic acid biosynthesis especially paculobutrazol (Gebre et al., 2012; Plaza-Wüthrich et al., 2016).

**Figure 1:**
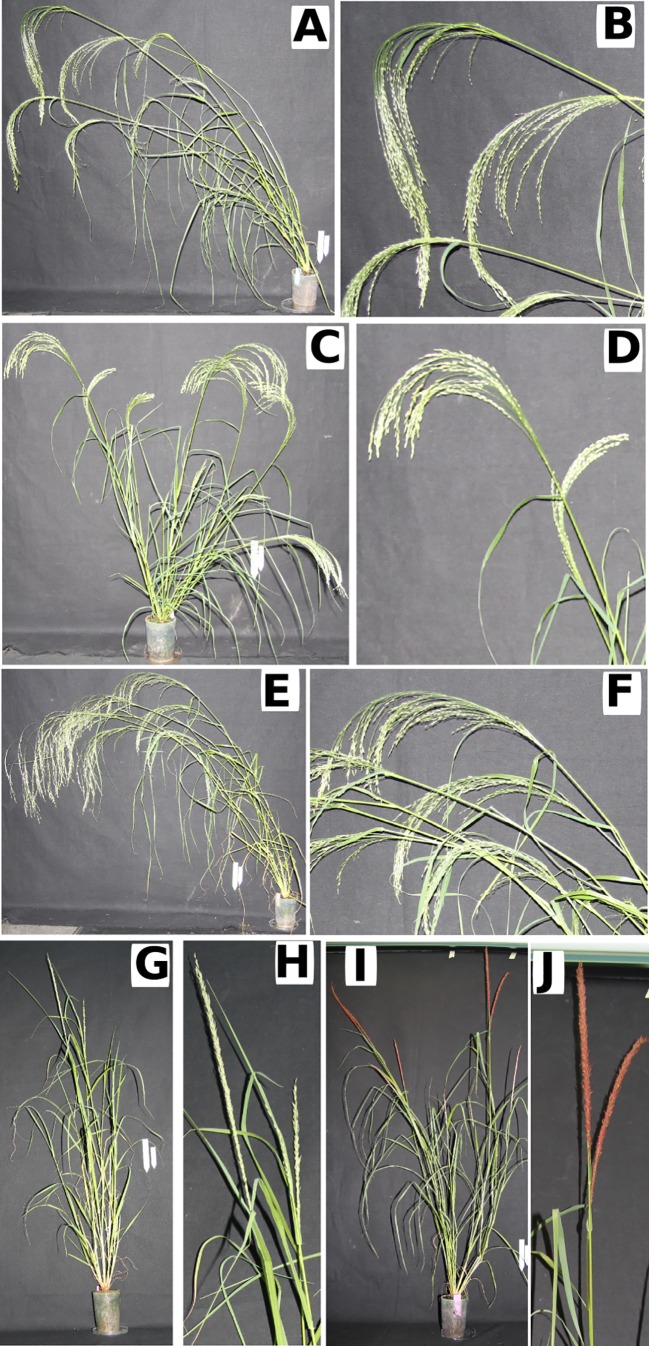
The tef genotypes differ in their overall morphology and the panicle morphology. **(A**, **B)** *Dukem*; **(C**, **D)** *Gommadie*; **(E**, **F)** *kinde*; **(G**, **H)** *Key Murri*; **(I**, **J)** *Quncho*. **(A**, **C**, **E**, **G**, **H)** Whole plant, **(B**, **D**, **F**, **H**, **J)** close up of panicles. Scale bar 10 cm.

In order to be successful, tef improvement projects must also take into account the agroecology and socioeconomic conditions of the region (Cannarozzi et al., 2018). We, therefore, compare morphological traits and measure mechanical properties in five different tef genotypes to investigate alternatives to breed for shorter stems. The mechanical and morphological data were combined to determine the likelihood of the stem breaking and introduce in a finite element model in order to better understand how they contribute to stem bending and the likelihood of the seeds touching the ground. There exists a huge diversity in tef genotypes in Ethiopia. We selected five tef genotypes for this study (**Figure 1**), two improved varieties; *Quncho* and *Dukem*, two landraces; *Gommadie* and *Key Murri* and a line generated from a mutant screen; *kinde*. *Quncho* tef variety is very popular in Ethiopia, mainly due to its high grain yield and a white seed color preferred by consumers (Assefa et al., 2011a). This variety was obtained by crossing two improved varieties (*Dukem* and *Magna*). *Dukem* is known for its high grain yield, but its pale white seed colour is least preferred by consumers. *Key Murri* and *Gommadie* are landraces collected from tef growing areas in Ethiopia. Since these two landraces have not undergone scientific improvement, their grain yield remains low. However, both *Key Murri* and *Gommadie* possess a compact panicle while other genotypes used in the current study have a loose panicle. Studies in rice have shown that panicle architecture can impact lodging (Xu et al., 2005; Wu et al., 2011). The inclusion of these two genotypes in the current study enabled us to investigate the effect of panicle morphology on the mechanical properties of tef plants. *Kinde* is a mutant line identified from an ethyl-methane sulfonate (EMS) mutagenized population by screening for gibberellic acid (GA) insensitivity (Cannarozzi et al., 2018) since GA insensitive mutants in wheat were semidwarf (Peng et al., 1999). Similar to GA insensitive mutants in other plants, *kinde* mutants are small in stature.

## Materials and Methods

### Plant Materials and Growing Conditions

Five tef lines with diverse morphological structures were used in the current study. *Dukem* and *Quncho* were obtained from the Ethiopian Institute of Agricultural Research while *Gommadie* and *Key Murrie* from USDA Agricultural Research Service through National Plant Germplasm System. *Kinde* is a mutant line developed at the Crop Breeding and Genomics lab in the Institute of Plant Sciences, University of Bern in Switzerland.

Seeds were grown in growth rooms (12-h light and 12-h dark, 12°C–20°C, the relative humidity was 80%). The soil which consisted of five parts of topsoil, four parts of turf and two parts of quartz sand was autoclaved before use. A Hauert Plantaktiv 16 + 6 + 26 type K fertilizer (Hauert HBG Dünger Schweiz, Grossaffoltern, CH) which contains 16% nitrogen, 6% phosphate, 26% potassium, 2% magnesium and micronutrients (0.02% borate, 0.04% copper, 0.1% iron, 0.05% manganese, 0.01% molybdenum, and 0.01% zinc) was applied weekly. For all experiments, the two main stems were taken per plant.

### Height and Weight of Plant Parts

Plant height was measured from the base at the junction between shoot and root to the tip of the plant while panicle length was quantified from the base to the tip of the panicle. Shoot biomass refers to the dry biomass of the individual plant at maturity, whereas panicle weight refers to the weight of panicle from each plant. All plant and panicle length and biomass related parameters were measured in the maturity of the plant.

### Lodging Angle

Lodging angle of tef plants was determined by measuring the angle from the tip of the panicle, in all panicles that were flowering using ImageJ (Schindelin et al., 2015). This angle is defined by the line from the stem-root border to the tip of each panicle and the vertical for each panicle individually. The angle of stems with immature panicles was also measured in the same way for use in the model.

### Internode Properties

Following the bending test, cross-sections of the internodes were taken close to the location of bending. Each internode of the main stem was measured and weighed. Following the mechanical testing, internodes were cross-sectioned close to the location of bending, and high-resolution images of the cross-sections were obtained with a binocular microscope in order to measure the inner and outer diameters of the hollow ellipse-shaped beam of tef internodes. The area, length, and mass of each internode were measured to compute the density of the different internodes for the model and the center of gravity. Not all plants belonging to a genotype had the same number of internodes. A small number of individuals had one more internode compared to the majority. In order to be able to build a model of a representative plant, from each genotype, it was necessary to assume that all plants had the most common number of internodes and the data from the additional internode was excluded. The following numbers of individuals had an extra internode for which data was not included: *Dukem* 2/45, *Gommadie* 2/60, *kinde* 23/70, *Key Murri* 2/83, *Quncho* 0/81.

### Mechanical Testing

Mechanical parameters were analyzed using a home-made three-point bending setup. The force was measured using a Futek 10-lb load cell (FSH03875, FUTEK Advanced Sensor Technology, USA), the displacement was performed using a Zaber robot (T-SR150B, Zaber Technologies Inc., Canada) as shown in **Supplementary Figure S1**.

To assess the mechanical strength of the culm, internodes on the main stem were cut, and each internode was tested using a standard three-point bending test. The travel distance was 10 mm, with an indentation step of 0.1 mm and a speed of 1.3 µm/ms. A force/displacement graph was simultaneously recorded and used to extract the mechanical properties of the stem section.

#### Flexural rigidity

The flexural rigidity (EI, Nm^2^) is the measure of the stiffness of the internode section (Van Delden et al., 2010):

$$\text{EI}=L^{3}(\text{dF}/\text{dY})/48$$

(dF/dY is the maximum initial slope of the force/deflection curve (N m^−1^). The Young's modulus (E, Nm^-2^), as a measure for material elasticity of the internode:

E = EI/I; where I is the second moment of area (m^4^) of a hollow, ellipse-shaped beam given by this equation: I = π/4 × (A^3^ × B – a^3^ × b); where A and a are the large diameters (m) of the outer and inner ellipse, respectively and B and b, the short diameters (m) of the outer and inner ellipse, respectively (m). The large diameters are perpendicular to the force direction applied in the three-point bending test.

The following mechanical properties were measured based on (Crook and Ennos, 1994) and used in tef by (Van Delden et al., 2010):

Self-weight moment: The self-weight was computed using the following formula:

Ms = sin (theta) × h × m × g where theta is the angle of the plant, h is its height of the center of gravity of the plant, m its mass and g is the force due to gravity. The center of gravity is computed from the height of the internode × mass of internode × total mass^-1^.

#### Safety Factor

The safety factor was computed using the following formula:

SF = Ss/Ms; where Ss = Fmax × L × 0.25, where L is the distance between the supports in the three-point bending experiment (Van Delden et al., 2010).

### Statistical Analysis

Statistical analysis was performed using the aov function in R followed by Tukey's Honest Significant Difference test. To compare high versus low lodging species a Mann-Whitney-Wilcoxon Test was performed, using the wilcox.test function in R. For the Principal Component study, statistical analysis was done in the R programming environment following (Wehrens, 2011; Piccolo, 2018) using the built-in function prcomp which does principal component analysis. This was used to investigate the relationship between the following variables: lodging angle (angles), plant height, center of gravity (CG), mass, panicle angle (P_angle), panicle weight (P_weight), the self-weight moment (MS), the self-weight moment if lodging angle is fixed at 10° (MS10), Fmax, EI, SS, SF (safety factor), SF10 (safety factor if the lodging angle is fixed at 10°), cross-sectional area and thickness of the cortex. The data was log-transformed and scaled to standardized values (mean = 0, variance = 1).

### Model

The finite element method simulations were performed in Abaqus/CAE 6.12-1 standard/explicit (Abaqus, 2018). The tef plant was approximated as a series of cylinders representing the different internodes. A 3D deformable shell sweep was used to construct the part. The path is defined using the length of the internodes measured for the individual genotypes. The section is defined using the average dimension of the cross-section of the tef stem. The material is defined as elastic and isotropic with the Young’s modulus measured from the three-point bending experiments. The Poisson ratio was set to zero. The thickness of the material was measured from cross-sections of each internode. The internode density was computed from the mass of each internode divided by the volume as computed from the cross-sectional area and the length of the internodes. A force of gravity was added to the total model at 10 N. The stem was initially given a small bend corresponding to the angle of the immature stems. The angle of the panicle was measured in the individual genotypes. The final internode representing the panicle was put at the measured angle from the stem. Apart from the weight and length, the panicle was not measured, so the cross-section and E were set to be the same as for the final internode before the panicle.

## Results

### A Comparison of the Morphological Properties of Different Tef Lines

#### A Comparison of Lodging Tolerance in Different Tef Lines

**Figure 2:**
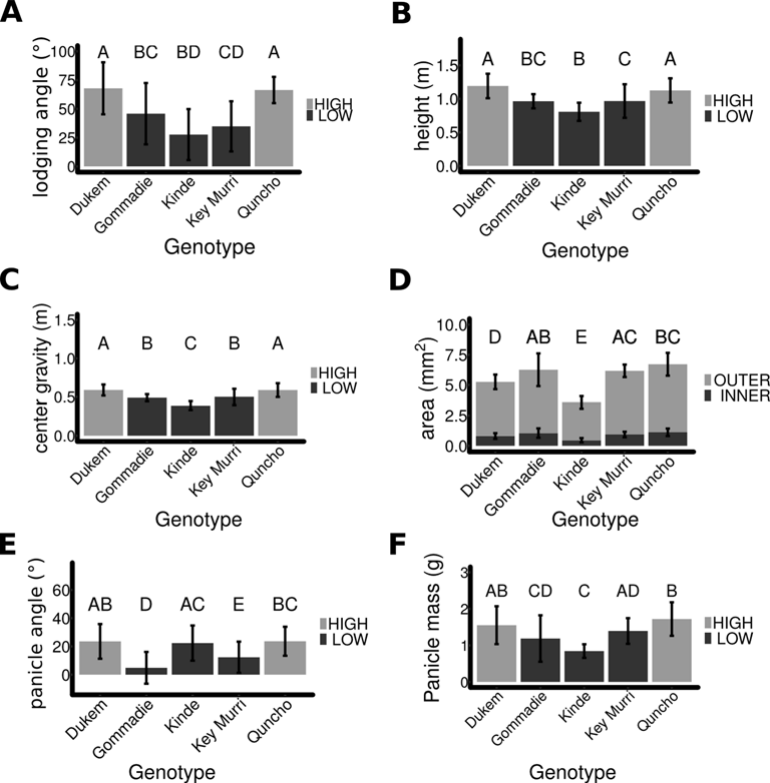
A comparison of morphological traits in different tef genotypes. **(A)** Lodging angle in the different Tef genotypes. **(B)** Plant height of the five genotypes. **(C)** The center of gravity of each ecotype determined by measuring the mass and length of each internode. **(D)** The cross-section of the first internode of each genotype. The area of the hollow inside (dark) was subtracted from the outer area (light). **(E)** The angle between the tip of the panicle and stem. **(F)** The mass of the panicle. Bars show mean error for standard deviation. The same letter denotes sample means that are not significantly different from each other (p > = 0.05) as determined by Tukey HSD.

Lodging tolerance of tef plants was determined by measuring the angle of the whole plant at flowering time (**Figure 2A**). The lines could be separated into two significantly different groups as determined by a Wilcox rank sum test (p < 0.0001): those with a high lodging angle *Dukem* (68° ± 23 SD) and *Quncho* (66° ± 11 SD) and those with a lower lodging angle *Gommadie* (46° ± 27 SD), *Key Murri* (35° ± 22 SD) and *kinde* (28° ± 22 SD). The susceptibility to lodging might be related to the type of panicle the plant possesses. While the two improved genotypes with high lodging angle (*Dukem* and *Quncho*) have loose panicle, the two landraces with low lodging angle (*Gomaddie* and *Key Murri*) possess compact panicles (**Figure 1**). Although the *kinde* mutant has a loose panicle, its lodging angle is similar to those of genotypes with compact panicles.

#### Plant Height and Center of Gravity for the Different Lines

The height of each plant was measured (**Figure 2B**). The average height of the high lodging genotypes was significantly higher (1.17 m ± 0.18 SD, n = 72), compared to the low lodging genotypes (0.93 m ± 0.20 SD, n = 81) (p < 0.0001). This indicates that in addition to the panicle type, the height of the plant also affects the severity of lodging in tef plants. In order to determine the center of gravity of the plants (**Figure 2C**), the weight and length of each internode were measured (**Supplementary Figures S2A, B**). The center of gravity was significantly higher for the high lodging varieties (0.6 m ± 0.09 SD, n = 79) than the lower lodging ones (0.48 m ± 0.09 SD, n = 85) (p < 0.0001). This shows that the values of the center of gravity are associated with susceptibility of the plant to lodging. The higher the center of gravity, the higher the lodging incidence.

#### Stem Cross-Section Area

The cross-section of each internode was measured for each genotype (**Supplementary Figures S2C**). The cross-section of the first internode which is most critical for culm-breaking type lodging did not differ significantly (p > 0.1) between the high and low lodging genotypes (**Figure 2D**); however, *kinde* and *Dukem* had a significantly smaller cross-section. The relationship between the cross-section of the first internode and the lodging angle could not be observed since *Quncho*, an improved variety with high lodging angle is grouped together with *Gommadie* and *Key Murri*, the two natural accessions with low lodging angle. The extremely low cross-section area (both the hollow and solid) in *kinde*, a mutant line with semidwarf stature but increased lodging tolerance showed that the size of stem cross-section does not explain the lodging tolerance. This is especially true for the first bottom internode.

#### Panicle Angle and Weight

The angle between the tip of the panicle and the main stem was measured in a subset of plants (**Figure 2E**). It was significantly higher in the higher lodging lines (23° ± 11, n = 244 panicles) compared to the lower lodging lines (15° ± 13, n = 291 panicles). An exception to this is the *kinde* mutant line which had a large panicle angle similar to the two high lodging genotypes (*Dukem* and *Quncho*). This is mainly due to the type of panicle. The three genotypes with loose panicles which also includes *kinde* had a high panicle angle while the remaining two genotypes (*Dukem* and *Quncho)* with compact panicles had small panicle angles. The weight of the panicle was also measured (**Figure 2F**) and was on average higher in the high lodging plants (1.63 g ± 0.5, n = 72) than the lower lodging plants (1.22 g ± 0.5, n = 81, p < 0.0001).

### A Comparison of Mechanical Properties in Different Tef Lines

**Figure 3:**
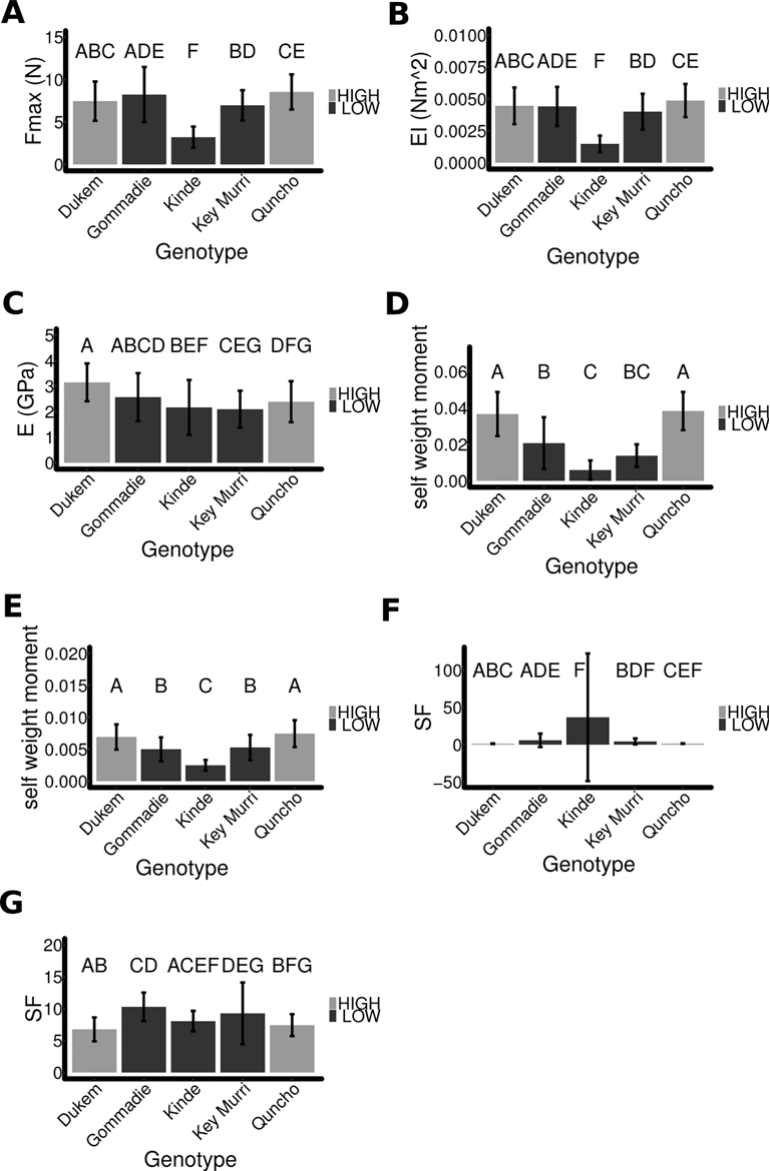
A comparison of the different mechanical properties of the tef ecotypes. **(A)** The breaking force of the first internode is shown. **(B)** The flexural rigidity was extracted from the three-point bending experiments. **(C)** The Young's modulus does not differ significantly between the high and low lodging ecotypes. **(D**, **E)** The self-weight moment of each ecotype was computed using **(D)** the measured angle and assuming **(E)** and angle of 10° for all species. **(F)** The safety factor of each ecotype was computed from the breaking force and the self-weight of the stem. The final angle of the mature stem was used. **(G)** The safety factor was computed assuming that all genotypes had the same angle of 10°. The same letter denotes means that are not significantly different with p > = 0.05 and determined using ANOVA, Tukey HSD.

In order to determine the resistance of the different tef genotypes to breaking, three-point bending tests were performed. The breaking force (Fmax) was measured for each internode of each genotype (**Figure 3A** and **Supplementary Figure S2D**). The breaking force was highest in the internodes at the base of the tiller and lower towards the top for all of the genotypes. The internode which usually breaks in culm-breaking lodging was internode one, the Fmax of internode one was significantly lower for the low lodging varieties due to the Fmax being significantly lower for *kinde* (3.2 N ± 1.2 SD, n = 18) compared to the other genotypes, and did not differ significantly between the other genotypes (7.7 N ± 2.3 SD, n = 146 plants). Thus, breaking strength is not sufficient to determine if a tef line will lodge.

The flexural rigidity of the different genotypes was compared by using the initial slope of the three-point bending experiment (see Methods) only *kinde* had a significantly (p < 0.0001) different flexural rigidity being much lower (0.00148 Nm^2^ ± 0.00065 SD, n = 18 plants) than that of the other genotypes (0.0044 Nm^2^ ± 0.0014 SD, n = 135 plants) (**Figure 3B** and **Supplementary Figure S2E**).

The Young's modulus is a measure of elasticity of a material. It was computed from the flexural rigidity by taking into account the area of the stem. The Young's modulus was significantly slightly lower for the low lodging varieties (p < 0.001), but there was significant overlap between the individual genotypes (**Figure 3C** and **Supplementary Figure S2F**).

### A Comparison of the Safety Factor of the Different Tef Genotypes

As no single factor can predict whether a tef plant will lodge or not, we combined the factors to look at the result of the interaction. The self-weight moment (MS) is used to estimate the forces acting on the plant due to its own weight. MS increases as the plants start to lean over (Crook and Ennos, 1994).

We computed MS for the different genotypes, with theta being equal to the final stem angle (**Figure 3D**). The self-weight moment of the high and low lodging varieties was significantly different from each other (high lodging genotypes 0.037 Nm ± 0.011, low lodging genotypes 0.014 Nm ± 0.01, n = 72/81, p < 0.0001). This means that there was a higher force acting to break the stem and displace the root of the plant. The self-weight moment depends upon the angle that the plant has. In order to determine the impact of other factors, we investigated what the safety-factor would be if all the genotypes had the same arbitrary angle (theta = 10°). When all plants had the same angle the high and low lodging varieties still differ significantly (**Figure 3E**) (high lodging genotypes 0.0072 ± 0.002, low lodging genotypes 0.0046 ± 0.002, n = 72/81, p < 0.0001). This demonstrates that the different genotypes have different properties that contribute to their self-weight moment, and the previous result was not a consequence of them having already lodged.

The safety factor (SF) indicates how many times the stem can bear its own weight. The safety factor was computed for the different genotypes using the final angle that the mature plants had (**Figure 3F**). The lower lodging genotypes had a higher safety factor (11.97 ± 42, n = 72) compared to the higher lodging varieties (1.37 ± 42, n = 81). The safety factor depends on the angle of the plant and is extremely high at low angles. This was the case for some *kinde* plants and resulted in a highly variable result. If *kinde* is excluded, the lower lodging varieties still have a higher safety factor (4.8 ± 6.2, n = 63). As for the self-weight moment, we also compared the safety factor if the plants had the same arbitrary angle of 10° (**Figure 3G**). In this case, the high lodging varieties had a significantly lower safety factor (7.1 ± 1.8 SD, n = 72) compared to the low lodging varieties (9.3 ± 3.8 SD, n = 81; p < 0.0001). Individually, *kinde* was not significantly different from *Dukem* or *Quncho*, and *Key Murri* did not differ significantly from *Quncho*. This suggests that a major factor controlling the safety factor of *kinde* was the plant angle, i.e., stem bending type lodging.

### Modeling the Angle of Different Tef Genotypes

**Figure 4:**
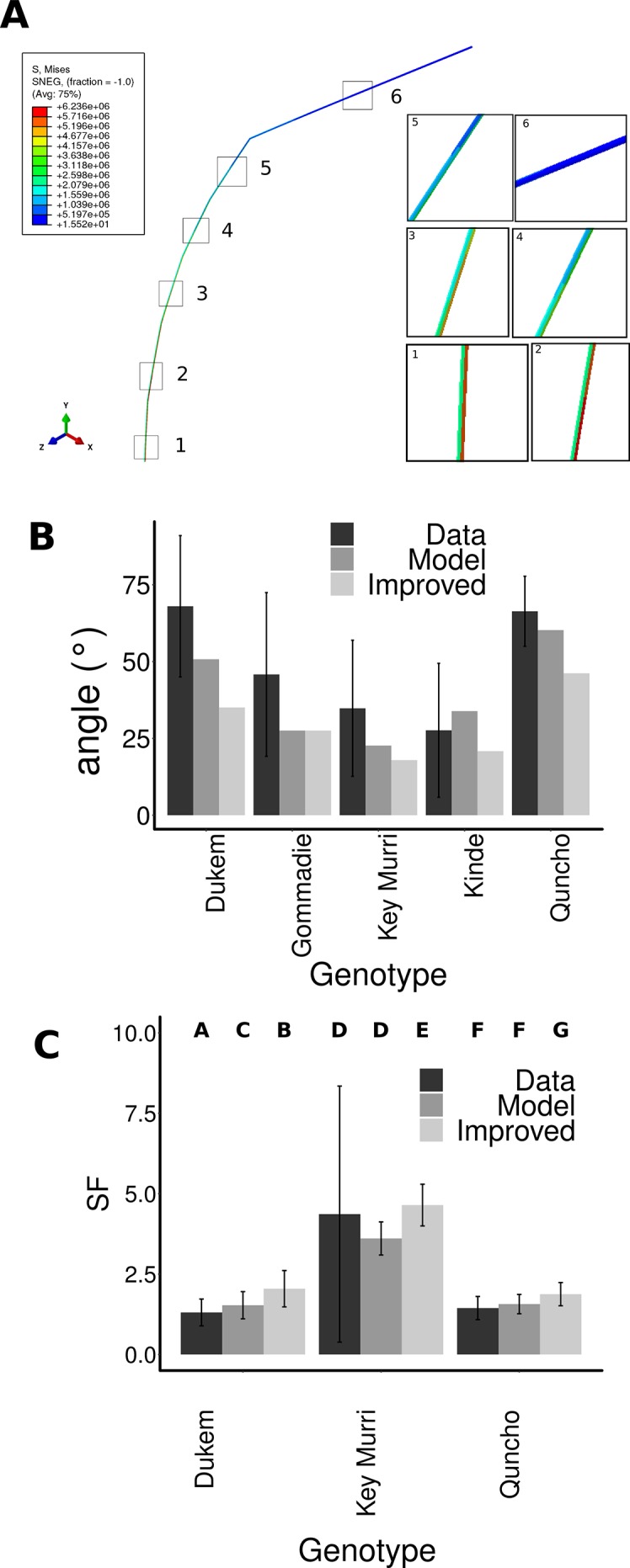
Modeling the lodging angle of different tef genotypes. **(A)** A simple finite element model was constructed, where each internode had the length, mass and elasticity. Gravity was applied to the model, and the base was prevented from moving or rotation. The stress was highest in the lower internodes one and two and lower in the higher internodes three to six. **(B)** The initial angle of the model was set to the angle of the immature stem before the flower emerges. The final angle that the model obtained under the force of gravity was measured (Model), and compared to the measured angles from the flowering stems (Data). The panicle angle of all genotypes was changed to be that of *Gommadie* (Improved). This led to an improvement of the lodging of all genotypes especially *Dukem* and *Quncho*. **(C)** The safety factor was compared for the genotypes using the lodging angle as extracted from the data, the model and using the improved model using the *Gommadie* panicle angle.

In tef, it is not only important if the stem breaks, but also if seed-bearing panicle touches the ground and spoil. Therefore, as well as contributing to the likelihood of breaking, the angle of the plant from the vertical is a very important factor in its own right. In order to determine why the plants had different angles that we observed, we built a simple finite element model of the different tef genotypes. The tef plants were approximated as a series of cylinders representing each internode. The length, density, elastic modulus, diameter and thickness were set according to the measured values (**Supplementary Figures S2A–H**). The starting condition for the model was one in which the stem had the angle of a tef stem before the flower emerges. This angle was distributed equally over the internodes. Gravity was set to act on all internodes. The model was secured at the base and thus only considered stem bending not root lodging. The panicle was put at the angle that the panicle was observed to have, as we did not have data on the mechanics of the panicle. The model was allowed to bend based on the weight of the internodes and gravity acting upon them. The final angle between the tip of the panicle and the vertical position was measured. We observed that for all genotypes, the highest stress was predicted to be on the bottom two internodes (**Figure 4A**, **Supplementary Figure S3**). Although this model is simple and does not account for the dynamics of the leaves hanging from the stem, the bending of the panicle, or the rotational stiffness of the root it is able to capture the lodging angle observed for four out of the five genotypes (*Quncho, Key Murri, kinde, and Gommadie*) (**Figure 4B**). The lodging angle of *Dukem* was higher than the model would predict. This may suggest that in *Dukem* root lodging is also contributing to the lodging angle. For the other ecotypes, the weight of the stem and its properties seems to be a good predictor of its lodging angle.

### Investigating the Parameters That Have the Biggest Impact on the Lodging Angle

In order to determine the most effective focus for future breeding programs, we performed a parameter search to determine which factor(s) had the most effect on the lodging angle. The panicle angle was associated with lodging likelihood; we determined the change in lodging if all genotypes had the panicle angle of *Gommadie*, but were otherwise the same (**Figure 4B**). This single change would reduce the predicted lodging angle from 51° to 35° in *Dukem* and from 60° to 46° in *Quncho*. The lodging angle of *kinde* could be improved from 34° to 21°, and that of *Key Murri* from 23° to 18°. This would result in a significant improvement in the safety factor of *Dukem* 2.05 ± 0.56 compared to the model using the *Dukem* panicle angle 1.53 ± 0.42 and the actual safety factor of the data 1.31 ± 0.42. In *Quncho*, the safety factor would improve from 1.4 ± 0.3 (data), or 1.56 ± 0.30 (model) to 1.88 ± 0.36. No significant improvement in the safety factor of *kinde* could be observed, likely owing to the large variability in the data (**Figure 4C**).

**Figure 5:**
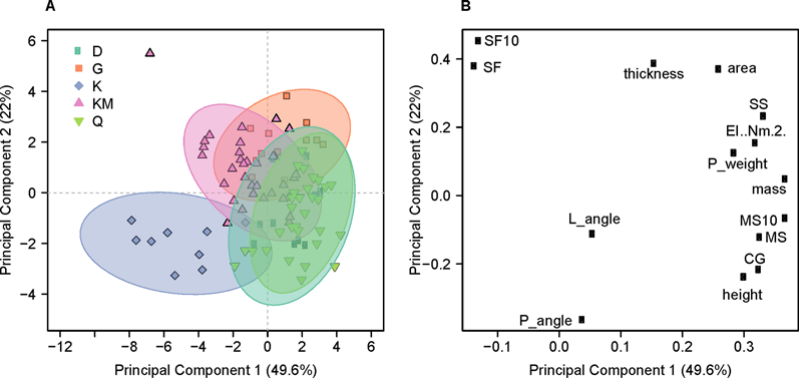
Principal Component Analysis for the top two components. **(A)** Scores of the first two principle components colored by genotype. *Quncho* (Q): green; *Dukem* (D): teal; *kinde* (K): purple; *Key Murrie* (KM): pink; and *Gommadie* (G): orange are shown for the 95 observations which had measurements for both panicle angle and panicle weight. Significant overlap is seen for *Quncho* and one of its parents, *Dukem*. *Kinde* is separated on both principle components. **(B)** Loadings of the first two principle components. Panicle angle (P_angle) is highly correlated with lodging angle (L_angle). In a correlation circle plot, the angle formed by any two variables and the origin reflects their pairwise correlation.

To further build on the findings of the model, we performed Principle Component Analysis (PCA) on the measured traits in order to evaluate their contribution to lodging angle. PCA is a dimension reduction technique used to investigate the relationships between 15 variables using 95 observations on five genotypes. The 15 variables investigated were: lodging angle, plant height, center of gravity, mass, panicle angle, panicle weight, the moment weight, the moment weight normalized to a 10-degree lodging angle, Fmax, EI, SS, SF (safety factor), SF10 (safety factor if the lodging angle is fixed at 10°), cross-sectional area, and thickness of the cortex. The data were log-transformed, which resulted in greatly reduced residuals and scaled. Because the panicle angle was only available for a limited number of observations, the analysis was done with 95 observations (**Figure 5**).

For the 95 observations that included panicle angle, the first principle component (PC1) explained 48% of the variance in the dataset while PC2 explained 22%. The scores plot shows that *kinde* forms a mostly separated cluster and a large amount of overlap between *Quncho* and one of its parents, *Dukem* (**Figure 5**). On the scores plot, PC1 separates *Dukem* and *Quncho* from *kinde*, while the loading plot shows that PC1 separates the mass-based variables (MS, CG, panicle mass, mass, etc.) from panicle angle, lodging angle and safety factor. PC2 separates *Gommadie* and *Key Murrie* from *kinde* and has extreme values for safety factor on one side and panicle angle and height on the other. The angle created by connecting any two variables with the origin shows the amount of correlation between the two variables. In support of the results from the modeling, the variable most correlated with lodging angle is panicle angle.

## Discussion

In this study, we compared the morphological and mechanical properties of five tef genotypes. We found that the higher-yielding improved genotypes such as *Quncho* and *Dukem* were more susceptible to lodging compared to the natural landraces such as *Key Murri* and *Gommadie*. We found that these genotypes differed more in terms of their morphological traits than their mechanical properties. This is mainly due to their differences in panicle form. *Quncho* and *Dukem* possess loose panicles, which covers a larger surface area that leads to them drooping down. The angle of the panicle causes the plant to be more liable to lodging. On the other hand, *Key Murri* and *Gommadie* possess compact or semicompact panicle types which stand erect and therefore do not pull the plant over. In field conditions, the impact of having a loose versus compact panicle may be stronger. A plant with a loose panicle is more likely to interact with plants in close proximity compared to plants with a compact erect panicle. This interaction would make the loose panicle plants even more liable to lodging. Hence, the lodging by compact panicle genotypes is substantially lower than those with loose panicle types.

In maize, it has also been demonstrated that stresses in the stalk are more sensitive to geometric factors than material properties (Von Forell et al., 2015). This suggests that morphology is a better focus for future breeding.

In comparison to other crops, tef has received less intense improvement. Tef has been reported to have a much lower safety factor when compared to other species (Van Delden et al., 2010). In our study we found that the high lodging varieties had a safety factor comparable to those identified for tef by Van Delden et al. (2010); however, the lower lodging varieties had a higher safety factor more similar to the one reported for winter wheat (Crook and Ennos, 1994). In wheat breeding, semidwarf plants are associated with reduced lodging. We saw that plant height and center of gravity did associate with lodging tolerance in tef. However, due to the high value of the tef straw as a livestock feed, breeding for a significant reduction in plant height might have little acceptance by the farmers (Yami, 2013). In order to be successful tef improvement projects must also take into account the agroecology and socioeconomic conditions of the region (Cannarozzi et al., 2018) and as such alternatives to shortening the stem must be found. The lower lodging varieties had a lower self-weight moment which makes them less likely to bend under their own weight. This is due in part to their shorter stature but also due to the reduced panicle weight. However, reducing panicle weight is likely to decrease grain yield and is also unpopular. In rice improvements have been seen by increasing the stem diameter and strength (Kashiwagi et al., 2008; Ookawa et al., 2010). We did not see a big difference in diameter between the high lodging and low lodging varieties in our study.

In comparison to tef and rice, wheat does not have a drooping panicle and is therefore predicted to behave differently (Van Delden et al., 2010). The two landraces used in the current study had compact panicles compared to the two improved varieties with loose panicle types. We made a simple model of each of the species and found that changing the panicle angle so that it remained more upright would reduce their stem lodging angle and increase their safety factor. Having an upright panicle is associated with having a more compact panicle such as we saw for the natural land races. *Kinde* which was isolated from the mutagenized population (Cannarozzi et al., 2018) for its semidwarf stature showed less lodging despite its loose panicle, likely owing to its small size. Lodging therefore seems to be controlled by panicle angle and plant height. However, having a more upright panicle seems to be a more attractive alternative as it maintains straw yields. Further work is needed to model the panicle and to examine fully its contribution to lodging. We, therefore, propose that future studies should focus on breeding for compact or semicompact panicle types. Studies in rice found that longer panicles reduced photosynthetic efficiency as a result of stem bending and had lower yields compared to compact panicles (Xu et al., 2005; Wu et al., 2011). Currently, the tef genotypes with compact panicles produce lower grain yield. This study suggests that it would be advantageous to produce high yielding lines with compact panicles. This work is currently underway with the development of a new line *Tesfa* (Kebede et al., 2018). *Tesfa* is a cross between *kinde* and *Key Murri*. It has a medium height and a compact panicle. This line has high yield, is lodging tolerant, has been approved for release (Cannarozzi et al., 2018), and is now being tested in field trials. There is scope to improve tef further by identifying the genes responsible for erect panicle architecture as has been achieved in rice (Xu et al., 2010) and using them for future breeding efforts.

Our study was made in the growth chambers where a number of environmental factors were controlled (the condition in the growth rooms was indicated in the Materials and Methods). Hence, this condition does not represent the field condition where a number of these variables are not controlled. It is important to note that this study did not include the effects of wind and rain which substantially affect the lodging of tef plants. However, we found that tall tef plants with a loose panicle are more likely to lodge, and in a field this is likely to be worse as these plants would have a tendency to occupy a larger surface area, which will make them more susceptible to wind, to push neighboring plants or to be pushed by those close to them.

In addition to stem lodging and stem bending which were the focus of this study, root lodging is also an important contributor to lodging in tef (Van Delden et al., 2010; Hirano et al., 2017). As root lodging also depends upon the self-weight moment, reducing the weight and having a more compact panicle will likely also reduce root lodging. The lower Young’s modulus found in many of the low lodging varieties while not be beneficial in reducing stem lodging may help to reduce root lodging as stronger stems in wheat have been blamed for increased root lodging (Crook and Ennos, 1994). Further work is needed to investigate the other factors that impact root lodging such as root properties and soil shear strength.

The PCA analysis using 95 observations grouped *kinde* separately from other four genotypes in the study. This is not unexpected since *kinde* with its semidwarf stature is distinctly different from the other four tall genotypes. The overlap observed here between *Quncho* and *Dukem* is expected since both of these genotypes are similar in plant height (tall), panicle type (loose), and susceptibility to lodging (very weak). This shows that PCA is efficient in grouping the five genotypes under investigation according to the overall architecture of the plant and resistance to the mechanical force of lodging. The PCA analysis supports the finding of the modeling that panicle angle is an important contributor to lodging angle.

## Conclusions

Tef is a vital crop in terms of its resilience to a wide range of environmental constraints and it provides nutritious and healthy food to the diet of a large population in the Horn of Africa. However, the plant heavily suffers from poor productivity which is mainly due to the weak mechanical force of the plant that cannot tolerate wind and rain. The novel biomechanical model of culm lodging presented here suggests that the angle of the panicle contributes to lodging in tef. Varieties with compact panicles tend to have a more favorable panicle angle. Compact panicles and reduced height contribute to lodging resistance but achieving this ideotype must be balanced with the need for a high yield of grain and straw.

## Data Availability Statement

The raw data supporting the conclusions of this article will be made available by the authors, without undue reservation, to any qualified researcher.

## Author Contributions

RB, SP-W, ZT: conceived the idea and designed the experiment. RB, SP-W, AW: performed the lab experiment. SR, PB, A-LR-K, GC: analyzed data. SR: made the model. SR, GC, ZT: wrote the paper. All authors read and approved the manuscript.

## Conflict of Interest

The authors declare that the research was conducted in the absence of any commercial or financial relationships that could be construed as a potential conflict of interest.

## Abbreviations

EBI, Ethiopian Biodiversity Institute; EMS, ethyl-methane sulfonate; GA, gibberellic acid; PCA, Principle Component Analysis; PRI, Pushing Resistance of Lower Part; SBI, Shortened Basal Internodes; SD, standard deviation; SF, safety factor; SMOS1, Small Organ Size 1.
