# Supplementary material for: Panicle Angle is an Important Factor in Tef Lodging Tolerance
Source: Front Plant Sci. 2020 Feb 13;11:61. doi: 10.3389/fpls.2020.00061 (PMC7031273; doi:10.3389/fpls.2020.00061)
Supplement: Supplementary file 1 [file Presentation_1.pdf]

Supplementary materials

Panicle angle is an important factor in tef lodging tolerance

Regula Blösch[1,*], Sonia Plaza-Wüthrich[1,*], Pierre Barbier de Reuille[1], Annett Weichert[1], Anne-Lise Routier-Kierzkowska[1,2], Gina Cannarozzi[1], Sarah Robinson[1,3,†], Zerihun Tadele[1,†]

[1]University of Bern, Institute of Plant Sciences, Altenbergrain 21, 3013 Bern, Switzerland

[2]University of Montreal, Institut de Recherche en Biologie Végétale, 4101 Sherbrooke East, Montréal, QC H1X 2B2, Canada

[3] The Sainsbury Laboratory, University of Cambridge, 47 Bateman Street, Cambridge CB2 1LR, United Kingdom

[*]These authors contributed equally to this work.

[†]Co-correspondence: sarah.robinson@ips.unibe.ch; Zerihun.tadele@ips.unibe.ch Telephone: + 41 31 631 4956

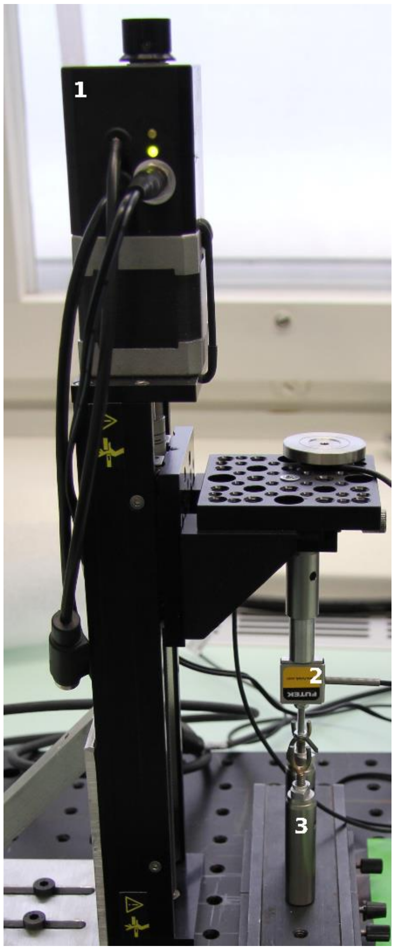

Supplementary Figure S1 The custom 3-point bending setup. 1) Zaber robot used to control and measure movement, 2) Futek load cell to measure force, 3) Support to hold sample.

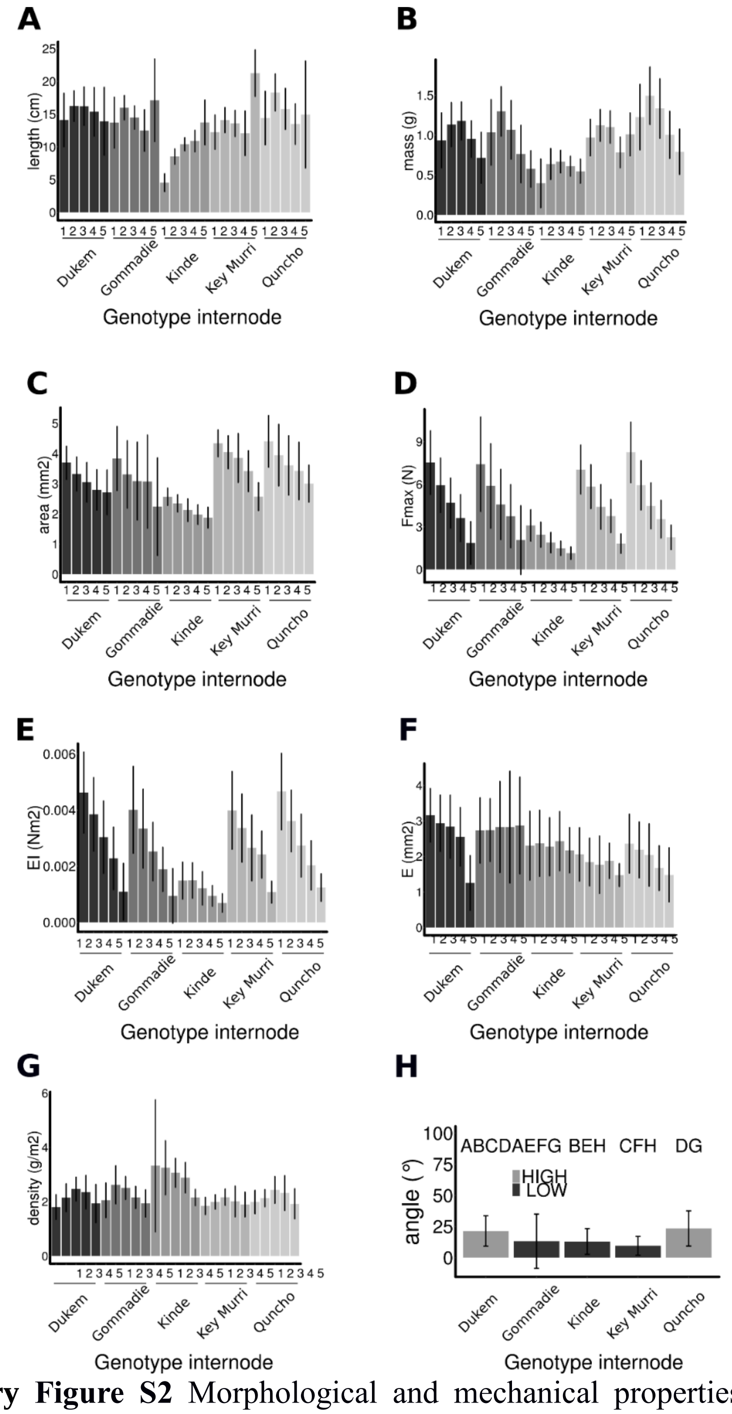

Supplementary Figure S2 Morphological and mechanical properties per internode from the different tef genotypes. The following properties were measured for each internode of the main stem of each ecotype. A) length, B) mass, C) cross-sectional area, D) breaking force, E) flexural rigidity, F) Youngs's modulus, G) the density computed from the length, area and mass, and. H) the angle of immature stems. The culm used for these studies contained flowers that had not fully emerged.

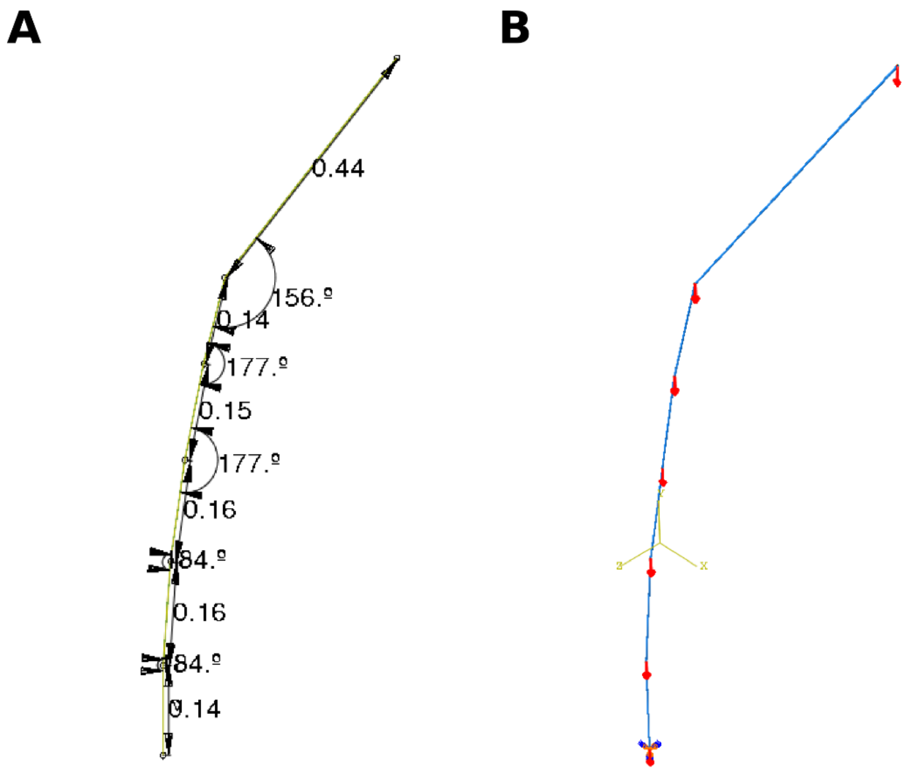

Supplementary Figure S3 Diagram of the model. A) The length of the internodes was set according to the measured values. The angle between each internode was the average of the angle of the pre-flowering stems. The angle between the panicle and the main stem was measured in post-flowering stems. B) The model was secured at the bottom to prevent translation or rotation. A force of 10 N was applied to the entire model to represent gravity.
